# HE3286, an oral synthetic steroid, treats lung inflammation in mice without immune suppression

**DOI:** 10.1186/1476-9255-7-52

**Published:** 2010-10-30

**Authors:** Douglas Conrad, Angela Wang, Raymond Pieters, Ferdinando Nicoletti, Katia Mangano, Anna M van Heeckeren, Steven K White, James M Frincke, Christopher L Reading, Dwight Stickney, Dominick L Auci

**Affiliations:** 1 VA San Diego Healthcare System, 3350 La Jolla Village Dr., San Diego, CA 92161, USA; 2 IRAS-Immunotoxicology, Utrecht University, P.O. Box 80176, 3508 TD Utrecht, The Netherlands; 3 Department of Biomedical Sciences, School of Medicine, Via Androne 83, 95124, University of Catania, Catania, Italy; 4 Case Western Reserve University, School of Medicine, Pediatric Pulmonology, 10900 Euclid Avenue, Cleveland, OH 44106-4948, USA; 5 Harbor Biosciences, 9171 Towne Centre Drive, Suite 180, San Diego, CA 92122, USA

## Abstract

**Background:**

17α-Ethynyl-5-androsten-3β, 7β, 17β-triol (HE3286) is a synthetic derivative of an endogenous steroid androstenetriol (β-AET), a metabolite of the abundant adrenal steroid deyhdroepiandrosterone (DHEA), with broad anti-inflammatory activities. We tested the ability of this novel synthetic steroid with improved pharmacological properties to limit non-productive lung inflammation in rodents and attempted to gauge its immunological impact.

**Methods and Results:**

In mice, oral treatment with HE3286 (40 mg/kg) significantly (*p *< 0.05) decreased neutrophil counts and exudate volumes (~50%) in carrageenan-induced pleurisy, and myeloperoxidase in lipopolysaccharide-induced lung injury. HE3286 (40 mg/kg) was not found to be profoundly immune suppressive in any of the classical animal models of immune function, including those used to evaluate antigen specific immune responses *in vivo *(ovalbumin immunization). When mice treated for two weeks with HE3286 were challenged with *K. pneumoniae*, nearly identical survival kinetics were observed in vehicle-treated, HE3286-treated and untreated groups.

**Conclusions:**

HE3286 represents a novel, first-in-class anti-inflammatory agent that may translate certain benefits of β-AET observed in rodents into treatments for chronic inflammatory pulmonary disease.

## Introduction

Chronic obstructive pulmonary disease (COPD), a term most often used to describe chronic bronchitis and emphysema [1,2] is an inflammatory disease of the lungs marked by a loss of elastic recoil, an increased resistance to airflow and decreased expiratory flow rate leading to dyspnea [3]. Chronic bronchitis, emphysema and cystic fibrosis (CF), all forms of COPD, share many features including a progressive airway remodeling driven by chronic inflammation [4-7]. COPD is a major cause of morbidity and mortality in industrialized countries and novel treatments are urgently needed because many patients respond poorly to conventional therapies [8-10]. Even in responders, narrow therapeutic windows and a myriad of unwanted side effects, including immune suppression are treatment limiting [9-12]. We have suggested that suitable agents may be found within the adrenal metabolome [13].

Dehydroepiandrosterone (DHEA) is an abundant adrenal steroid and a precursor in the biosynthesis of androgens, estrogens and other anti-inflammatory immune regulating steroids [14,15]. From studies reporting aberrant metabolism of adrenal steroids in CF patients [16,17] we surmised that novel anti-inflammatory therapeutics relevant to lung inflammation might be found within the DHEA metabolome. A large body of literature reports that DHEA replacement therapy (in animals, especially rodents) provides striking therapeutic benefits across a wide range of disease models [18]. However, DHEA replacement therapy in humans repeatedly failed to provide the same benefits observed in rodents [19-21]. Failures are attributed to poor (~3%) oral bioavailability, and a differential metabolism between rodents and humans that leads to different dominant downstream metabolic species [22-25]. Rodents rapidly metabolize exogenous DHEA into a surprisingly complex array of highly oxygenated metabolites [26-28]. We hypothesized that these metabolites may be responsible for activities attributed to DHEA [13].

Androstene-3*β*, 7*β*, 17*β-*triol (*β-*AET) is biosynthesized from DHEA, biologically active in rodents [29-32] and naturally occurring in humans [33-37]. It's functions in the body may include tissue-specific modulation of glucocorticoid (GC) action, immune function, and control of acute and chronic inflammation [38-40]. Despite these promising properties, *β*-AET suffers from some of the same pharmaceutical liabilities as DHEA, including metabolic instability and low oral bioavailability. Extensive screening studies demonstrated that HE3286, a synthetic derivative of *β*-AET, possessed surprising pharmaceutical properties including good oral bioavailability in rodents, primates and humans and significant resistance to steroidogenic metabolism, as evidenced by studies using human microsomes (Harbor Biosciences, unpublished observations). HE3286 also possessed anti-inflammatory properties, providing benefit in several animal models of immune-mediated inflammatory diseases [41-43]. In this report, we explore the potential of HE3286 for the treatment of lung inflammation using the murine models of carrageenan-induced pleurisy and LPS-induced lung injury. Immunological safety was assessed in the CFTR^-/- ^mouse model of CF, ovalbumin immunization, and in survival kinetics of mice challenged with lethal doses of the common lung pathogen, *Klebsiella pneumoniae*. The present studies, in context of our previous reports, suggest that HE3286 might also provide safe and effective treatment for patients with inflammatory diseases of the lung.

## Materials and methods

### Drugs

The test compounds HE3286 (17α-ethynyl-5-androstene-3β, 7β, 17β-triol), HE2000 (16α-bromoepiandrosterone) and vehicles (HERF405 or HERF202) were prepared and provided by Harbor Biosciences (San Diego, CA). HERF202 contains 30% β-cyclodextrin sulfobutyl ether sodium salt (w/v) in water. HERF405 contains 0.1% carboxymethylcellulose, + 0.9% NaCl + 2% Polysorbate 80 + 0.05% phenol. HE3286 was dissolved in HERF202 or suspended in HERF405 and administered by oral gavage and by subcutaneous injection (SC), respectively.

### Animal Care

Animals were purchased and housed in accordance with respective institutional guidelines and requirements of the relevant regulatory agencies. All studies were approved by the relevant institutional ethics committees. Pleurisy studies were performed by F.N. at University of Catania, Italy; LPS induced lung injury studies were performed by D.C. and A.W. at Veteran Affairs San Diego Medical Center; CFTR knockout mice studies were performed by A.V. at Case Western University, Cleveland, OH. Ovalbumin immunization and popliteal lymph node assays were performed by R.P at Utrecht University, and bacterial challenge studies were performed at Explora Biolabs (San Diego, CA)

### Carrageenan (CAR) -induced pleurisy mouse model

#### Animals

Six to 8 week old CD1 male mice (Charles River, Calco, Italy) were housed in a controlled environment and provided with standard rodent chow and water. All animals weighed approximately 25-30 grams each. These mice were acclimated for at least 3 days prior to the start of the experiment.

#### Experimental groups

Mice (n = 10 per group) were allocated into one of the following groups as follows: (1) Sham (saline) treated animals; (2) CAR only (CAR group); (3) CAR and vehicle (HERF405 by SC injection); (4 and 5) CAR and HE3286 (SC injection of either 40 or 4 mg/kg in vehicle); and (6) CAR and rabbit anti-mouse polyclonal anti-TNFα antibody (200 μg in saline, IP injection). All treatments were given 24 h and 1 h prior to CAR in a final volume of 0.1 mL.

#### Pleurisy Assay

Mice were anaesthetized with isoflurane and the skin was incised at the level of the left sixth intercostal space. The underlying muscle was dissected and saline (sham) or saline containing 2% λ-CAR (Sigma-Chimica, Milan, Italy) was injected into the pleural cavity. The skin incision was closed with a suture and the animals were allowed to recover. At 4 h after the injection of CAR, the animals were sacrificed by CO_2 _asphyxiation. The chest was carefully opened and the pleural cavity rinsed with 1 mL of saline solution containing heparin (5 U/mL) and indomethacin (10 μg/mL). The exudate and washing solution were removed by aspiration and the total volume measured. Any exudate, which was contaminated with blood, was discarded. The amount of exudate was calculated by subtracting the volume injected (1 mL) from the total volume recovered. The leukocytes in the exudate were suspended in phosphate-buffer saline (PBS) and counted with an optical microscope in a Burker's chamber after vital Trypan Blue staining. No differential cell counts were conducted, as cells at this time point are predominantly neutrophils [44]. Data are expressed as mL exudate volume or millions of neutrophils per mouse +/- standard deviation

### LPS -induced lung injury model

#### Animals

Six to 8-week old C57 black/6 male mice (approximately 25-30 grams, Harlan, San Diego, CA) were used in these studies (at least 4-8 animals per group). These mice were acclimated for at least 3 days prior to the start of the experiment. The animals were housed in a controlled environment and provided with standard rodent chow and water.

#### Chemicals and Reagents

LPS was prepared from *Escherichia coli *055:B5 (Sigma, St. Louis, MO)pou. Myeloperoxidase (MPO) enzymatic activity was assessed as previously described [45]. TNFα and IL-6 EIA kits were purchased from Assay Designs (Ann Arbor MI).

#### Lung injury model

Animals were treated with HE3286 or with vehicle (HERF405) *via *a single gavage administration (0.1 mL) 24 h and 1 h before LPS challenge. LPS challenge was performed by lightly anesthetizing the mice with isofluorane, and then directly administering the LPS (5 mg/kg, 50 μL; 1 mg diluted in 1 mL sterile saline) into the trachea under direct observation with a gel loading pipette through a medical otoscope. The mice were placed in a vertical position and rotated for 0.5 - 1 min to distribute the instillate evenly within the lungs. At 48 h after the LPS challenge, animals were sacrificed, bronchoalveolar lavage (BAL) samples taken (BAL performed 3× using sterile PBS; 1.3 mL were typically recovered) cells counted using a hemacytometer and cytokine levels were measured by ELISA.

### Ovalbumin mouse immunization studies

Female BALB/c mice (5 per group) were sensitized by intraperitoneal injection (total volume 0.2 mL) on days 1 and 8 with 100 μg ovalbumin (endotoxin-free OVA from Sigma Aldrich, Zwijndrecht, the Netherlands) precipitated with aluminum hydroxide (Sigma Aldrich) in saline. Mice were treated (gavage) daily with HE3286 (40 mg/kg) or with vehicle (HERF202) on days 0-20. On day 20, 2 h after the final treatment, blood was drawn by terminal cardiac puncture, serum prepared and tested by ELISA for antibody titres against OVA. Briefly, OVA was coated overnight (4°C) on 96 well plates (high bond 3950 Costar plates, Cambridge MA) in carbonate buffer (pH 9.6), and then blocked with PBS-Tween 20/3% milk powder (Campina melkunie, the Netherlands) for 1 h at 37°C. Serum diluted in PBS-Tween 20 (0.5%) was incubated in the wells for 1 h, followed with incubation (1 h, 37°C) with alkaline phosphatase-conjugated anti-IgG1 antibodies (Southern Biotechnology Association Inc., Birmingham, USA). Subsequently, 1 mg/mL p-nitrophenylphosphate in diethanolamine buffer was used for the color reaction, which was stopped with an EDTA solution. Absorbance at 450 nm was measured using an ELISA reader (ELX800, Biotek Instruments-Inc, Winooski).

### *Klebsiella pneumoniae *survival study

#### Animals

Female BALB/c mice (approximately 25-30 grams, Harlan, San Diego, CA) were used in these studies. Mice were acclimated for at least 3 days prior to the start of the experiment.

#### Challenge

Animals were randomized by weight into 4 groups. Group 1 (n = 10) received daily 0.1 mL administrations (gavage) of HE3286 at 80 mg/kg in vehicle (HERF405). Group 2 (n = 10) received equal volumes of vehicle alone. Group 3 (n = 10) received daily IP administrations of dexamethasone (dex; 0.4 mg/kg, Sigma, St. Louis, MO) in 0.1 mL saline. Group 4 (n = 8) was untreated. Body weights were measured daily. After 14 days of treatment, infection was induced by subcutaneous inoculation of 10^7 ^colony-forming units (LD_50 _at 72 hours; Harbor Biosciences, unpublished observations) of *K. pneumoniae *(strain AFRRI7). Once daily treatments were given until death. All animals were monitored twice-daily for health status until the end of the study.

### Studies in CFTR knockout mouse model

#### Animals

STOCK *Cftr^tm1Unc^*-TgN(FABPCFTR)#Jaw were bred, housed and used as in our previous studies [46,47]. Male mice (9 per group) 6-8 weeks of age, body weight at least 16 g, were used in these experiments and bred and housed under standard laboratory conditions.

#### Infection model

The slow growing mucoid clinical strain *P. aeruginosa *M57-15 was used in these studies. *P. aeruginosa*-laden agarose beads were made and used, as described previously [46,48] with minor differences. Mice were inoculated with a 1:35 dilution of the beads. (LD_50 _dose). HE3286, HE2000 (0.1 mL) or vehicle (HERF202) was given by oral gavage 24 h before and 1 h after bacterial challenge. Measurements of bacterial burden in the lungs were performed as in our previous studies [49].

### Statistical Analysis

For pleurisy studies, all parameters of interest were subjected to ANOVA with Duncan's new multiple-range *post hoc *testing between groups. For lung injury studies, data were analyzed by two-sided Student's *t *test. For CFTR knockout mouse studies, data were analyzed by one-way ANOVA and stratified Mann-Whitney. For OVA immunization studies, analysis was performed using the SAS^® ^system, (version 9.1) with certain exact tests implemented by use of the StatXact^® ^(version 7) software package [50]. For *Klebsiella pneumoniae *survival studies, comparison of survival curves (Logrank test for trends) was performed using Prism software (San Diego, CA).

## Results

HE3286 and HE2000 appeared well tolerated throughout the course of these studies. No drug related frank toxicity (i.e., animals found dead or in moribund condition) or unexpected weight loss was observed in any of the treated animals as compared to vehicle controls (data not shown).

### Effect of HE3286 in carrageenan-induced pleurisy mouse model

**Figure 1:**
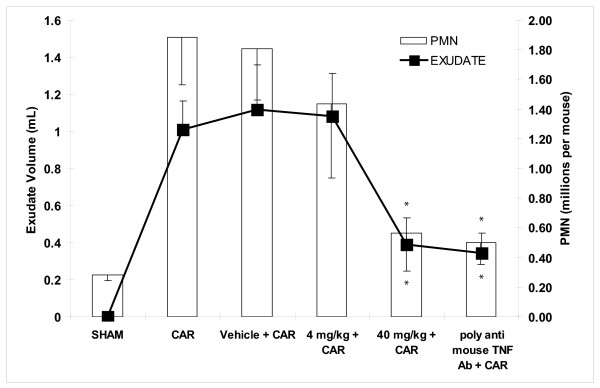
**Effect of HE3286 treatment on carrageenan-induced pleurisy**. Mice CD1 mice (10 per group) were anesthetized and saline (0.1 mL) alone (sham) or saline containing 2% carrageenan (CAR) was injected into the pleural cavity. Mice were treated (sc) with HE3286 (4 or 40 mg/kg) or vehicle HERF405 alone (0.1 mL) 24 h before and 1 h before CAR. At 4 h after CAR, the animals were killed, the chest opened, and the pleural cavity rinsed with 1 mL of saline solution. The leukocytes in the exudate were suspended in phosphate-buffer saline (PBS) and counted. Data are expressed as mL exudate volume (A) or millions of neutrophils (B) per mouse +/- standard deviation on the Y-axis. Treatment groups are identified on the X-axis. *p < 0.05.

When mice were challenged with 0.l mL of 2% carrageenan in the pleural cavity, high leukocyte numbers (~1.9 × 10^6 ^per mouse) were observed in the pleural exudate. Substantially lower leukocytes numbers (~2.8 × 10^5 ^per mouse) were observed in animals undergoing a sham procedure and challenged with saline (Figure 1). When mice were pre-treated with HE3286 (40 mg/kg) by subcutaneous injection, significantly (*p *< 0.05) reduced numbers of carrageenan-induced neutrophils (~5.7 × 10^5^) were observed in pleural exudates compared to those observed in animals given vehicle alone (~1.8 × 10^6^). The 4.0 mg/kg dose was not effective. Treatment with high-dose HE3286 was as effective as treatment with polyclonal anti-mouse TNFα antibody, positive control. Treatment with HE3286 also reduced pleural exudate volumes (compared to vehicle), in a dose-dependent fashion.

### Effect of HE3286 in the LPS-induced lung injury mouse model

**Figure 2:**
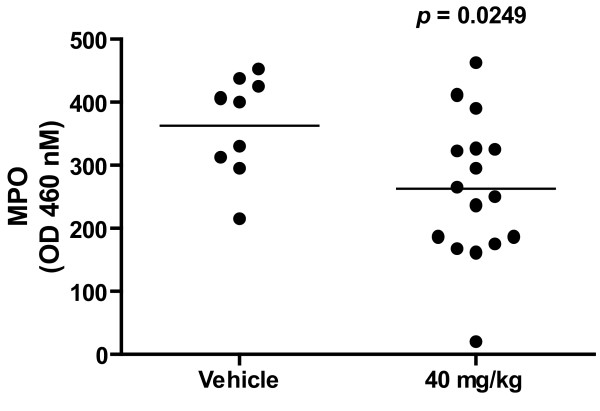
**Effect of HE3286 treatment on MPO levels in LPS induced Lung Injury**. On day-1, male C57 black/6 mice were pre-treated (gavage) with HE3286 or 0.1 mL vehicle (HERF405). The next day, mice were challenged with 50 μg of E-coli LPS under direct visualization of trachea under light anesthesia. Sixty minutes after the LPS challenge, mice were treated with a second dose of HE3286, or vehicle. Forty-eight hours after LPS challenge, mice were sacrificed and myeloperoxidase (MPO) activity in lungs determined as previously described [45]. Results are from two identical experiments. Data are expressed as O.D at 460 nM.

The ability of HE3286 to reduce lung inflammation was also tested in the LPS-induced acute lung injury model. A meta-analysis of two independent studies revealed that when mice pre-treated with HE3286 (40 mg/kg) by oral gavage were challenged with 50 mg of LPS, levels of MPO in lungs at 48 hours were significantly (*p *< 0.025) reduced (~30%) compared to vehicle-treated animals (Figure 2). Reductions in MPO were also observed with HE3286 at lower doses (12 and 1.2 mg/kg), but as with inflammatory cells and cytokines (TNFa and IL-6) in bronchoalveolar lavage fluid (BAL), upon meta-analysis, these changes did not achieve statistical significance (data not shown).

### Effect of HE3286 in the murine ovalbumin immunization model

**Figure 3:**
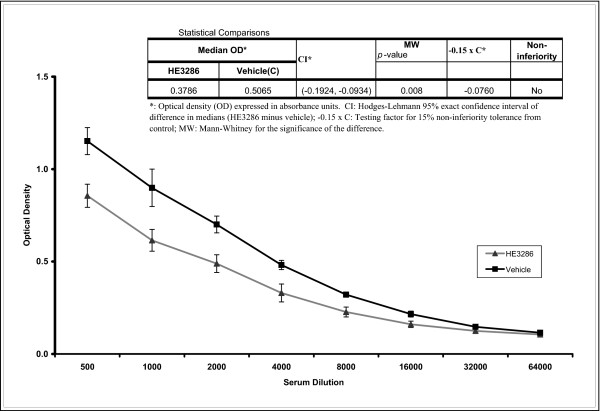
**Effect of HE3286 on OVA-specific immunoglobulin production**. Female BALB/c mice (5 per group) were sensitized by intraperitoneal injection (total volume 200 μL) on days 1 and 8 with 100 μg OVA precipitated with alum (25 mg/mL) in saline. Animals were treated (gavage) with HE3286 (40 mg/kg) or with HERF202 vehicle (100 μL) alone once daily for twenty days. On day twenty, animals were sacrificed and OVA-specific immunolglobulin (IgG) levels in serum were measured at various dilutions by ELISA. Data are expressed as optical density +/- standard deviation on the Y-axis versus dilution on the X-axis.

We have shown in previous studies that HE3286 does not suppress either delayed type hypersensitivity responses [51] or mixed lymphocyte responses [42], classical measures of cell mediated (i.e., Th1) biased immunity. HE3286 showed no suppressive activity or immune toxicity in the reporter antigen popliteal lymph node assay [51]. Immunization with ovalbumin in alum adjuvant is a classical approach to induce antibody (i.e., Th2) biased immune responses [52]. Profound immune suppression was not observed in the murine ovalbumin immunization model. However, a small (~25%) but statistically significant (*p *< 0.05) reduction in OVA specific antibody production was observed in mice treated with HE3286 (Figure 3). The statistical analysis shows that, in terms of derived IgG1 absorbance, HE3286 is inferior to its vehicle (*p *= 0.008). The exact confidence interval for the difference in median absorbance is negative, indicating that the distribution of HE3286 optical density is unlikely to be on a par with that of its vehicle.

In order to estimate the clinical relevance of the above finding and to assess the immunological impact of treatment with HE3286, studies in mice challenged with opportunistic lung pathogens were undertaken.

### Effect of HE3286 on opportunistic bacterial infections of the lung

A major limitation of GC treatment, and a potential advantage of HE3286, is that the former is immune suppressive and the latter is not. The following studies were designed to demonstrate this, especially in the context of opportunistic infections.

#### 1. *K. pneumoniae*

**Figure 4:**
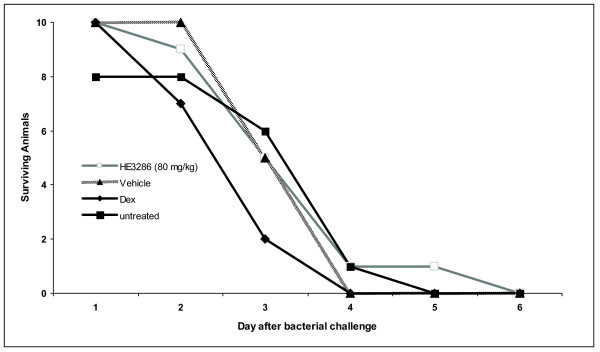
**Effect of HE3286 on bacterial infection**. Female BALB/c mice (n = 8-10 per group) received daily 0.1 mL administrations (gavage) of HE3286 at 80 mg/kg in vehicle (HERF405), equal volumes of vehicle alone, daily IP administrations of dexamethasone (dex; 0.4 mg/kg) in 0.1 mL saline or sham treated. After 14 days of treatment, infection was induced by SC inoculation of 10^7 ^colony-forming units (cfu) of *K. pneumoniae*. Daily HE3286 or dex treatments continued and all animals monitored twice-daily until the end of the study for health status.

*K. pneumoniae *is an opportunistic infection commonly observed in immune suppressed mice [53]. When animals were challenged with 10^7 ^cfu of K. *pneumoniae*, no significant differences in survival kinetics were found between HE3286-treated, vehicle-treated or untreated groups. Fifty to 60% of animals in these groups survived to day 3 (Figure 4). In contrast, in the dex-treated group, only 20% of animals were alive on day 3. This difference (*p *= 0.07 *vs *untreated) suggested that dex-treated animals succumbed to infection more quickly than controls. Mice treated with HE3286 appeared to gain more weight compared to other groups. At the time of bacterial challenge, there was a significant (*p *= 0.01) difference between control, dex-treated and HE3286-treated animals. After bacterial challenge, controls and dex-treated animals appeared to lose weight faster and to a greater extent than the HE3286-treated mice (data not shown).

#### 2. HE2000, but not HE3286, reduced bacterial burden in the CFTR mouse model

*P. aeruginosa *is another opportunistic bacterial pathogen that is commonly found resident in lungs of patients with CF [54]. In the context of the present studies, it was deemed important to demonstrate that HE3286 did not exacerbate bacterial burden in this COPD-like setting. In this study, another synthetic steroid, HE2000, was used as a positive control, to demonstrate that the bacterial burden delivered to these animals was indeed amenable to pharmacological manipulation. Neither HE3286 nor HE2000 (positive control) treatment induced frank toxicity in the CFTR^-^/^- ^mouse and there was no significant (ANOVA) difference between groups (vehicle *versus *drug-treated) with respect to body weight or bronchoalveolar lavage cell counts at 24 hours after bacterial challenge (data not shown). There was significantly greater numbers of bacteria in vehicle-treated mice compared to 40 mg/kg HE2000 (*p *< 0.03) as was found in our previous studies [55]. In contrast, we found no significance with respect to a reduction of bacteria in HE3286- compared to vehicle-treated mice (Table 1).

**Table 1:** Effect of HE3286 on bacterial burden in lungs of CFTR^-/- ^Mice

	CFU (in millions of units)		
**Group**	***n***	**Med (IQR)**	***p***

Vehicle	9	7.00 (4.15, 8.60)	.

HE3286 40 mg/kg	8	5.35 (3.08, 8.00)	0.8026

HE2000 40 mg/kg	9	3.60 (2.10, 4.80)	*0.0290*

Mice were inoculated with a 1:35 dilution of *P. aeruginosa*-laden agarose beads. HE3286, HE2000 (positive control) or vehicle alone was delivered by oral gavage (0.1 mL) to mice 24 h before and 1 h after challenge with bacteria laden beads. Bacterial colony counts were performed on whole lung homogenates taken 24 h after the final challenge. Data are expressed as CFU *per *lung.

CFU: colony forming units; *p*: Stratified Mann-Whitney exact *p*-value (versus vehicle); IQR: Interquartile rang; *n*: sample size.

## Discussion

We have shown that in rodent models of lung-associated inflammation, HE3286 acts as an anti-inflammatory steroid without clinically relevant immune suppression. HE3286 treatment reduced inflammation in carrageenan-induced pleurisy as judged by reduced numbers of neutrophils and pleural exudate volumes and in the LPS-induced lung injury model as judged by reduced MPO in BAL fluid. HE3286 treatment was safe in the CFTR mouse model (no observed increase in bacterial burden) and induced only slight suppression of antigen specific antibody production in the OVA immunization model. The limited adverse immunological impact of this latter observation was clearly demonstrated in animals treated (for 14 days) with HE3286 and then challenged with a lethal bacterial infection. These animals had similar survival kinetics as vehicle-treated and untreated mice. As expected, mice treated with the well-known immune suppressive agent dexamethasone succumbed to infection faster than either of the untreated groups.

The activity of HE3286 in pleurisy suggests a profound anti-inflammatory effect of HE3286 on the early events driving acute lung inflammation. Significant decreases in both neutrophils and exudate volumes were observed 4 hours after carrageenan challenge. HE3286 was also tested in two independent LPS-induced lung injury studies. The activity of HE3286 later in the acute inflammatory response (i.e., 48 hours after challenge) was most apparent in these studies when the compound was given at the highest dose (40 mg/kg). Meta-analysis of the two studies revealed significantly reduced levels of MPO in lungs and non-significant reductions in pro-inflammatory cells and cytokines in BAL. Lower doses of HE3286 appeared less effective since reductions did not reach statistical significance in our meta-analysis. Observations in BAL were likely limited by variability in the assay, its kinetics [56-58] and statistical limitations imposed by the small number of animals per study. Nevertheless, the preponderance of evidence in this model confirms an anti-inflammatory activity of HE3286 relevant to lung inflammation.

Our observations that HE3286 possesses significant anti-inflammatory activity in both carrageenan and LPS-induced lung inflammation are consistent with our earlier observations in models of rheumatoid arthritis [41,42], experimental autoimmune encephalitis and colitis [43]. We reported that oral HE3286 treatment significantly decreased disease scores in all models. In our rodent model studies of rheumatoid arthritis, we found that HE3286 treatment benefit was associated with reduced IL-17, TNFα and IL-6 signaling and dramatic reductions in IL-6 and matrix metalloproteinase mRNA in inflamed joint tissue accompanied by an expansion of regulatory T cells in the spleen [51]. Differential HE3286 dosing effects have been observed between the various rodent disease models. For example, in EAE, HE3286 was effective at 4 mg/kg [43], while in RA models, the minimally effective dose was 10 fold higher [41]. And in the rat model of colitis, 30 mg/kg was less effective than l0 mg/kg, suggesting that in specific instances, the compound may be more effective at lower doses [43].

The biological mechanism by which HE3286 mediates these effects is unknown. In our previous studies, benefit was associated with reduced activation of NFκB in splenocytes from LPS-challenged mice [51]. Evidence has accumulated implicating NFκB as a mediator of lung injury in rodents [59] and as a potential target for treating COPD [3,4]. These findings suggest HE3286 down-regulates NFκB-mediated pro-inflammatory cytokine production in the lungs. As in rodent models of rheumatoid arthritis, inhibition of matrix metalloproteinases may have also played a protective role in LPS-induced lung injury that is also characterized by a marked increase of MMP9 in the lung [59]. The implication of MMP3 in the tissue destruction associated with COPD [60] further highlights some of the immunopathogenic similarities of this disease with the LPS-induced lung injury model and highlights the potential relevance of these findings to the clinical setting. Regarding a possible mechanism for action of HE3286 through the TNFα pathway, in our previoius studies we found that HE3286 caused the inhibition of the LPS-induced macrophage activation program *in vitro *primarily by inhibiting TNFα action [61]. This activity was associated with significantly decreased phosphorylation of IKK, NFκB, P38, and JNK. HE3286 treatment was also associated with increased regulatory T cells. This same mechanism may also explain the HE3286 induced reduction of IgG1 we observed in our OVA studies.

Notably, HE3286 at the highest doses was not found to be immune suppressive in any of the classical *in vitro *(mitogen induced lymphocyte proliferation) or in *vivo *models (DTH, poplitieal lymph node assay, viral endocarditis) of immune suppression [42,51]. In the present studies, treatment resulted in a small but significant suppression of OVA specific antibody production. However, HE3286 was found to be safe in the *CFTR *^-/- ^male mouse model of cystic fibrosis. Further, *K. pneumoniae *challenge to animals conditioned with HE3286 resulted in no promotion of death. Therefore, our studies in both CFTR^-/- ^and *K. pneumoniae *challenged mice suggest no clinical relevance to this observation. We speculate that decreased levels of IL-6 in HE3286-treated animals may be causal to this phenomenon.

The molecular target of HE3286 remains unknown. HE3286 does not interact (either *via *binding or transactivation) with any of the known nuclear hormone receptors, including the glucocorticoid or sex steroid receptors [61]. Since no dedicated nuclear receptors have ever been identified for 7-hydroxy steroids, potential mechanisms of action have previously been grouped into four broad categories, including gating (ligand inactivation), modulation of ion channels, interaction with atypical receptors, and modulation of steroidogenic enzymes [38]. Potential HE3286 targets within each of these categories are currently under consideration. In tissues, HE3286 and/or metabolites may have multiple sites of interaction as is the case for other members of the steroid hormone series [62]. None of our observations rule out the possibility that metabolites of HE3286 significantly contribute to the anti-inflammatory activities and as such they must be considered as potentially relevant in a systems biology paradigm [63]. As a direct consequence, the pro-inflammatory disease process may be interrupted at multiple nodes through restoration of homeostatic endocrinology in the host.

HE3286 appears to ameliorate insulin resistance [64] and colitis [65], co-morbidities commonly associated with CF and other COPDs [66-68]. These pre-clinical observations have lead to clinical trials. Preliminary observations indicate that an anti-inflammatory activity of HE3286 has been demonstrated in obese insulin resistant subjects [61]. Taken together, the new data presented here suggest an even broader application for this agent in inflammatory conditions of the lung. HE3286 may represent a novel, first in class anti-inflammatory and disease-modifying agent that has a safety profile that permits chronic use without the side effects produced by the presently prescribed anti-inflammatory agents.

## Statement of competing interests

Employees of Harbor Biosciences hold equity positions in Harbor Biosciences. Harbor Biosciences funded the studies and financed publication of the manuscript. Harbor Biosciences holds patents related to HE3286.

## Authors' contributions

DC and AW carried out the lung injury studies. FN and KM carried out the pleurisy studies. RP carried out OVA immunization assay. AH carried out the CFTR mouse studies. SW, JF CR and JF participated in the design of the study, interpretation and performed or supervised the statistical analysis. DA conceived of the study, participated in its design and coordination, and drafted the manuscript. All authors read and approved the final manuscript.
